# Supplementary material for: Modeling glioblastoma heterogeneity as a dynamic network of cell states
Source: Mol Syst Biol. 2021 Sep 16;17(9):e10105. doi: 10.15252/msb.202010105 (PMC8444284; doi:10.15252/msb.202010105)
Supplement: Supplementary file 5 — Source Data for Figure 3 [file MSB-17-e10105-s001.zip › Figure3A_sourcedata/GSEA_3065/hallmarks_state2.GseaPreranked.1623416269049/HALLMARK_ESTROGEN_RESPONSE_EARLY.html]

Details for gene set HALLMARK\_ESTROGEN\_RESPONSE\_EARLY[GSEA]

|  || Dataset | state2 |
| Phenotype | NoPhenotypeAvailable |
| Upregulated in class | na\_neg |
| GeneSet | HALLMARK\_ESTROGEN\_RESPONSE\_EARLY |
| Enrichment Score (ES) | -0.41927883 |
| Normalized Enrichment Score (NES) | -1.6533598 |
| Nominal p-value | 0.0 |
| FDR q-value | 0.0072903633 |
| FWER p-Value | 0.043 |
Table: GSEA Results Summary

  

Fig 1: Enrichment plot: HALLMARK\_ESTROGEN\_RESPONSE\_EARLY      
 Profile of the Running ES Score & Positions of GeneSet Members on the Rank Ordered List

  

| PROBE | GENE SYMBOL | GENE\_TITLE | RANK IN GENE LIST | RANK METRIC SCORE | RUNNING ES | CORE ENRICHMENT || 1 | RBBP8 |  |  | 141 | 0.325 | 0.0250 | No |
| 2 | TUBB2B |  |  | 322 | 0.225 | 0.0335 | No |
| 3 | NXT1 |  |  | 326 | 0.224 | 0.0608 | No |
| 4 | PPIF |  |  | 580 | 0.166 | 0.0542 | No |
| 5 | RHOBTB3 |  |  | 612 | 0.162 | 0.0709 | No |
| 6 | CCND1 |  |  | 840 | 0.134 | 0.0631 | No |
| 7 | FKBP4 |  |  | 1079 | 0.113 | 0.0516 | No |
| 8 | ARL3 |  |  | 1127 | 0.110 | 0.0601 | No |
| 9 | FKBP5 |  |  | 1180 | 0.106 | 0.0676 | No |
| 10 | OLFM1 |  |  | 1263 | 0.101 | 0.0713 | No |
| 11 | MYBL1 |  |  | 1390 | 0.093 | 0.0692 | No |
| 12 | FARP1 |  |  | 1441 | 0.090 | 0.0750 | No |
| 13 | PDLIM3 |  |  | 1841 | 0.071 | 0.0410 | No |
| 14 | RAB31 |  |  | 1977 | 0.065 | 0.0346 | No |
| 15 | BAG1 |  |  | 2120 | 0.060 | 0.0269 | No |
| 16 | ADD3 |  |  | 2401 | 0.051 | 0.0031 | No |
| 17 | BCL2 |  |  | 2499 | 0.048 | -0.0014 | No |
| 18 | SIAH2 |  |  | 2599 | 0.045 | -0.0065 | No |
| 19 | ITPK1 |  |  | 2684 | 0.042 | -0.0103 | No |
| 20 | IL17RB |  |  | 2712 | 0.041 | -0.0080 | No |
| 21 | FDFT1 |  |  | 2717 | 0.041 | -0.0034 | No |
| 22 | SNX24 |  |  | 2784 | 0.039 | -0.0056 | No |
| 23 | ISG20L2 |  |  | 3387 | 0.026 | -0.0668 | No |
| 24 | SLC9A3R1 |  |  | 3823 | 0.018 | -0.1112 | No |
| 25 | MYC |  |  | 3842 | 0.018 | -0.1108 | No |
| 26 | REEP1 |  |  | 4225 | 0.012 | -0.1503 | No |
| 27 | RARA |  |  | 4228 | 0.011 | -0.1491 | No |
| 28 | GLA |  |  | 4388 | 0.009 | -0.1650 | No |
| 29 | NRIP1 |  |  | 4712 | 0.004 | -0.1991 | No |
| 30 | WWC1 |  |  | 4718 | 0.004 | -0.1992 | No |
| 31 | MYBBP1A |  |  | 4769 | 0.003 | -0.2042 | No |
| 32 | SEC14L2 |  |  | 4889 | 0.001 | -0.2168 | No |
| 33 | RRP12 |  |  | 5129 | -0.002 | -0.2421 | No |
| 34 | UNC119 |  |  | 5150 | -0.002 | -0.2440 | No |
| 35 | CANT1 |  |  | 5151 | -0.002 | -0.2437 | No |
| 36 | MED24 |  |  | 5189 | -0.003 | -0.2472 | No |
| 37 | ELF1 |  |  | 5246 | -0.004 | -0.2528 | No |
| 38 | KLF10 |  |  | 5308 | -0.005 | -0.2587 | No |
| 39 | AR |  |  | 5392 | -0.007 | -0.2668 | No |
| 40 | RPS6KA2 |  |  | 5397 | -0.007 | -0.2663 | No |
| 41 | INPP5F |  |  | 5513 | -0.009 | -0.2776 | No |
| 42 | AKAP1 |  |  | 5534 | -0.009 | -0.2786 | No |
| 43 | ABLIM1 |  |  | 5605 | -0.010 | -0.2849 | No |
| 44 | PMAIP1 |  |  | 5617 | -0.010 | -0.2848 | No |
| 45 | NADSYN1 |  |  | 5842 | -0.014 | -0.3071 | No |
| 46 | DYNLT3 |  |  | 5958 | -0.016 | -0.3174 | No |
| 47 | FASN |  |  | 6119 | -0.019 | -0.3323 | No |
| 48 | HES1 |  |  | 6191 | -0.020 | -0.3375 | No |
| 49 | MAPT |  |  | 6242 | -0.021 | -0.3403 | No |
| 50 | NAV2 |  |  | 6389 | -0.023 | -0.3530 | No |
| 51 | MREG |  |  | 6506 | -0.025 | -0.3623 | No |
| 52 | TMEM164 |  |  | 6524 | -0.026 | -0.3610 | No |
| 53 | ABAT |  |  | 6574 | -0.027 | -0.3629 | No |
| 54 | SCARB1 |  |  | 6752 | -0.031 | -0.3781 | No |
| 55 | SEMA3B |  |  | 6759 | -0.031 | -0.3750 | No |
| 56 | AFF1 |  |  | 6774 | -0.031 | -0.3726 | No |
| 57 | XBP1 |  |  | 6931 | -0.035 | -0.3850 | No |
| 58 | TIAM1 |  |  | 6948 | -0.035 | -0.3824 | No |
| 59 | BLVRB |  |  | 7000 | -0.036 | -0.3834 | No |
| 60 | ELOVL5 |  |  | 7020 | -0.037 | -0.3808 | No |
| 61 | MED13L |  |  | 7165 | -0.041 | -0.3912 | No |
| 62 | FOS |  |  | 7222 | -0.043 | -0.3919 | No |
| 63 | WFS1 |  |  | 7253 | -0.044 | -0.3897 | No |
| 64 | ABCA3 |  |  | 7275 | -0.044 | -0.3865 | No |
| 65 | SLC1A4 |  |  | 7280 | -0.044 | -0.3815 | No |
| 66 | FAM102A |  |  | 7294 | -0.045 | -0.3774 | No |
| 67 | NBL1 |  |  | 7312 | -0.045 | -0.3737 | No |
| 68 | TSKU |  |  | 7606 | -0.053 | -0.3984 | No |
| 69 | ADCY9 |  |  | 7646 | -0.055 | -0.3959 | No |
| 70 | SLC39A6 |  |  | 7769 | -0.059 | -0.4017 | No |
| 71 | TFAP2C |  |  | 7828 | -0.060 | -0.4004 | No |
| 72 | CA12 |  |  | 8005 | -0.067 | -0.4109 | Yes |
| 73 | NCOR2 |  |  | 8039 | -0.069 | -0.4060 | Yes |
| 74 | TIPARP |  |  | 8047 | -0.069 | -0.3982 | Yes |
| 75 | AMFR |  |  | 8048 | -0.069 | -0.3897 | Yes |
| 76 | SLC7A5 |  |  | 8067 | -0.070 | -0.3830 | Yes |
| 77 | TOB1 |  |  | 8169 | -0.076 | -0.3845 | Yes |
| 78 | UGCG |  |  | 8171 | -0.076 | -0.3753 | Yes |
| 79 | SH3BP5 |  |  | 8247 | -0.080 | -0.3735 | Yes |
| 80 | CXCL12 |  |  | 8251 | -0.080 | -0.3639 | Yes |
| 81 | FLNB |  |  | 8416 | -0.090 | -0.3704 | Yes |
| 82 | ENDOD1 |  |  | 8528 | -0.099 | -0.3701 | Yes |
| 83 | GJA1 |  |  | 8728 | -0.114 | -0.3773 | Yes |
| 84 | KDM4B |  |  | 8737 | -0.115 | -0.3640 | Yes |
| 85 | BHLHE40 |  |  | 8804 | -0.123 | -0.3559 | Yes |
| 86 | SYNGR1 |  |  | 8812 | -0.124 | -0.3413 | Yes |
| 87 | TPBG |  |  | 8829 | -0.127 | -0.3274 | Yes |
| 88 | MYOF |  |  | 8851 | -0.129 | -0.3137 | Yes |
| 89 | B4GALT1 |  |  | 8942 | -0.140 | -0.3061 | Yes |
| 90 | SLC16A1 |  |  | 8970 | -0.145 | -0.2911 | Yes |
| 91 | PRSS23 |  |  | 8975 | -0.145 | -0.2736 | Yes |
| 92 | SVIL |  |  | 8981 | -0.146 | -0.2561 | Yes |
| 93 | SLC26A2 |  |  | 9005 | -0.149 | -0.2401 | Yes |
| 94 | DHCR7 |  |  | 9009 | -0.150 | -0.2219 | Yes |
| 95 | CELSR2 |  |  | 9020 | -0.152 | -0.2042 | Yes |
| 96 | CHPT1 |  |  | 9057 | -0.159 | -0.1884 | Yes |
| 97 | IL6ST |  |  | 9090 | -0.164 | -0.1716 | Yes |
| 98 | IGF1R |  |  | 9152 | -0.178 | -0.1562 | Yes |
| 99 | LRIG1 |  |  | 9229 | -0.204 | -0.1391 | Yes |
| 100 | PODXL |  |  | 9234 | -0.205 | -0.1142 | Yes |
| 101 | ABHD2 |  |  | 9250 | -0.213 | -0.0895 | Yes |
| 102 | RETREG1 |  |  | 9262 | -0.218 | -0.0637 | Yes |
| 103 | SLC2A1 |  |  | 9349 | -0.272 | -0.0394 | Yes |
| 104 | CD44 |  |  | 9407 | -0.397 | 0.0035 | Yes |
Table: GSEA details [plain text format]

  

Fig 2: HALLMARK\_ESTROGEN\_RESPONSE\_EARLY: Random ES distribution      
 Gene set null distribution of ES for **HALLMARK\_ESTROGEN\_RESPONSE\_EARLY**
